# Animal-Associated Exposure to Rabies Virus among Travelers, 1997–2012

**DOI:** 10.3201/eid2104.141479

**Published:** 2015-04

**Authors:** Philippe Gautret, Kira Harvey, Prativa Pandey, Poh Lian Lim, Karin Leder, Watcharapong Piyaphanee, Marc Shaw, Susan C. McDonald, Eli Schwartz, Douglas H. Esposito, Philippe Parola, for the GeoSentinel Surveillance Network

**Affiliations:** Aix Marseille Université, Marseille, France (P. Gautret, P. Parola);; Institut Hospitalo-Universitaire Méditerranée Infection, Marseille (P. Gautret, P. Parola);; Centers for Disease Control and Prevention, Atlanta, Georgia, USA (K. Harvey, D.H. Esposito);; CIWEC Clinic Travel Medicine Center, Kathmandu, Nepal (P. Pandey);; Tan Tock Seng Hospital, Singapore (P.L. Lim);; Lee Kong Chian School of Medicine, Singapore (P.L. Lim);; Monash University, Melbourne, Victoria, Australia (K. Leder);; The Royal Melbourne Hospital, Parkville, Victoria, Australia (K. Leder);; Mahidol University, Bangkok, Thailand (W. Piyaphanee);; Travellers Health and Vaccination Centre, Auckland, New Zealand (M. Shaw);; University Hospital of Northern British Columbia, Prince George, British Columbia, Canada (S.C. McDonald);; Tel Hashomer and Sackler School of Medicine, Tel Aviv, Israel (E. Schwartz)

**Keywords:** rabies, animal-related exposure, travel, GeoSentinel, viruses, rabies, rabies virus

## Abstract

Among travelers, rabies cases are rare, but animal bites are relatively common. To determine which travelers are at highest risk for rabies, we studied 2,697 travelers receiving care for animal-related exposures and requiring rabies postexposure prophylaxis at GeoSentinel clinics during 1997–2012. No specific demographic characteristics differentiated these travelers from other travelers seeking medical care, making it challenging to identify travelers who might benefit from reinforced pretravel rabies prevention counseling. Median travel duration was short for these travelers: 15 days for those seeking care after completion of travel and 20 days for those seeking care during travel. This finding contradicts the view that preexposure rabies vaccine recommendations should be partly based on longer travel durations. Over half of exposures occurred in Thailand, Indonesia, Nepal, China, and India. International travelers to rabies-endemic regions, particularly Asia, should be informed about potential rabies exposure and benefits of pretravel vaccination, regardless of demographics or length of stay.

## Abstract 2

No demographic characteristics identified who might benefit most from pretravel counseling.

Rabies causes ≈60,000 human deaths annually and is a public health concern in most countries in Asia and Africa (*1*). By contrast, it is rare among travelers; an average of 3.7 cases were documented each year during 2004–2012 (*2*). Nevertheless, bites to travelers by potentially rabid animals are relatively frequent; estimated incidence is 0.4% per month of stay, according to a meta-analysis of ≈1,270,000 travelers (*3*). By inference, expensive postexposure prophylaxis (PEP), which includes administration of rabies vaccine and rabies immunoglobulin, is probably provided to large numbers of travelers annually. Given the severity of rabies virus infection and the high costs associated with caring for large numbers of potentially exposed travelers, rabies pretravel preventive measures need to be reinforced. These measures include systematic pretravel counseling about animal bite avoidance, postexposure wound care and prophylaxis, and preexposure rabies vaccination for some travelers.

Generalizability of data regarding the epidemiology of travel-associated animal-related rabies virus exposures are limited because they come from studies that are small or single center or that focus on travelers returning from specific destinations. As such, travelers at highest risk for rabies cannot be reliably identified on the basis of available data (*3*,*4*). The decision as to which travelers should receive predeparture rabies vaccination is complex because of the combination of limited data defining rabies risk among travelers, the high cost of rabies vaccine and rabies immunoglobulin in some countries, and the occasionally limited rabies vaccine and rabies immunoglobulin availability because of production problems. 

One way to assess the epidemiology of travel-associated illness in travelers and immigrants involves use of GeoSentinel, a global sentinel surveillance network established in 1995 through a collaborative effort from the International Society for Travel Medicine and the US Centers for Disease Control and Prevention (CDC) (*5*). We used the GeoSentinel database to assess geographic and demographic factors for a large number of patients who sought care at GeoSentinel sites for animal-related exposure (e.g., bite, scratch, lick on broken skin or mucous membrane) and required rabies PEP.

## Methods

### Data Source

GeoSentinel Surveillance participating sites are specialized travel or tropical medicine clinics in 24 countries on 6 continents; they systematically contribute point-of-care, clinician-based, sentinel surveillance data. Sites are staffed by clinicians recruited on the basis of their knowledge and experience in travel and tropical medicine (*6*). To be included in the database, patients must have crossed an international border within 10 years of the clinic visit and sought medical care for a presumed travel-related illness. Diagnoses are selected by the evaluating clinician from a standard list of ≈500 causative or syndromic diagnoses. Data about demographics, travel history, and presumed country of exposure are also collected. Region of travel is calculated from country of exposure by using the following modified regional groupings established by the United Nations Children’s Fund: Australia/New Zealand, Caribbean, Central America, Eastern Europe, Middle East, North Africa, North America, North East Asia, Oceania, South America, South Central Asia, South East Asia, sub-Saharan Africa, and Western Europe (*6*). Institutional review board approval was not required because the GeoSentinel data collection protocol was reviewed at CDC and classified as public health surveillance and not human subject research.

### Inclusion Criteria

We reviewed all records of patients who sought care at a GeoSentinel site from January 1, 1997, through December 31, 2012, and for whom data were entered into the GeoSentinel database. Analysis was limited to travelers with confirmed or probable final diagnoses of an animal exposure and receipt of rabies PEP. We excluded patients who reported animal exposure but did not receive rabies PEP (which probably includes those exposed to animals other than mammals as well as mammals in areas where rabies is absent) and patients who received rabies PEP but did not report animal exposure.

### Statistical Analyses

First, we conducted a descriptive epidemiologic analysis. Eligible records were stratified by exposure animal (dog, bat, cat, nonhuman primate [NHP], and other mammal). For patients seeking care after travel, duration of travel was calculated as the last day of the most recent trip minus the first day of the most recent trip. For patients seeking care during travel, travel duration was calculated as the date of the clinic visit subtracted from the trip departure date. Trips could have involved multiple countries; therefore, travel duration does not always represent time in the exposure country. Patients were excluded from this calculation if they did not list recent travel that included their country of exposure, if duration of travel could not be calculated or was invalid, or if they listed multiple trips to the country of exposure within the past 6 months.

Second, we conducted a subanalysis for temporal reporting trends in rabies risk exposure relative to total GeoSentinel reports among patients who received treatment during the final 10-year reporting period (2003–2012). For this analysis, we included only a subset of GeoSentinel sites contributing patient data for the entire 10-year period. A simple linear regression was used for this calculation. A 2-tailed p value of <0.05 was considered statistically significant. All analyses were performed by using SAS 9.3 (SAS Institute, Inc., Cary, NC, USA).

## Results

### Patient Characteristics and Animals Associated with Exposure 

The analysis included 2,697 patients who had received rabies PEP at 1 of 45 GeoSentinel sites after an animal-related exposure during 1997–2012. These patients represented 1.5% of the 183,749 ill travelers entered into the database during the same 16-year period. Nearly all (99%) patients who reported animal exposure were evaluated in the outpatient setting; most (74%) travelers sought care in their country of residence after return from travel, and the others (25%) sought care during travel at GeoSentinel clinics in or near a destination country. The most frequent region of residence was Western Europe (32%), followed by northeastern Asia (17%), Australia/New Zealand (17%), Southeast Asia (14%), and North America (10%); 8% had emigrated from their country of birth to another country. A pretravel encounter with a health care provider was recorded for 32% of patients, no pretravel consultation was reported by 42%, and this information was unknown or missing for 26%. Information about pretravel rabies vaccination status was available for 756 (28%) patients, 83 (11%) of whom were vaccinated before traveling.

The animal species associated with exposure was recorded for 2,637 (98%) patients (Table 1). The most common species were dog (60%) and NHP (24%), followed by cat (10%) and bat (2%). Among patients in this analysis, about half were male; however, male patients accounted for slightly more than half of the exposures to dogs and less than half to NHPs, cats, and other mammals. The median age of patients was 30 years (range birth–90 years). Overall, the proportion of children <15 years of age was 11%, but children were overrepresented among cat exposures (19%) and underrepresented among bat exposures (4%). The most common reason for travel was tourism (71%), followed by visiting friends and relatives (12%) and business (10%). Tourists made up a disproportionately large proportion (92%) of those exposed to NHPs.

**Table 1:** Characteristics of 2,697 patients who sought care for an animal exposure and received rabies postexposure prophylaxis at GeoSentinel Surveillance Network sites, January 1997–December 2012, by animal species*

Patient characteristic	Animal					
	Dog	NHP	Cat	Bat	Other†	Total‡
No. patients	1,618	638	271	46	126	2,697
Male sex, no. (%)	891 (55)	269 (42)	125 (46)	21 (46)	54 (43)	1,360 (51)
Age, y, no. (%)						
<14	160 (10)	65 (10)	50 (19)	2 (4)	14 (11)	291 (11)
15-44	1,027 (64)	460 (72)	151 (56)	28 (61)	75 (60)	1,739 (65)
45-64	340 (21)	103 (16)	56 (21)	16 (35)	33 (26)	548 (20)
>65	87 (5)	9 (1)	13 (5)	0	4 (3)	113 (4)
Reason for travel						
Tourism	1,016 (63)	590 (92)	183 (68)	31 (67)	89 (71)	1,908 (71)
Visiting friends/relatives	264 (16)	6 (1)	41 (15)	1 (2)	11 (9)	323 (12)
Business	206 (13)	18 (3)	25 (9)	2 (4)	13 (10)	264 (10)
Missionary/volunteer/researcher/aid worker	82 (5)	15 (2)	14 (5)	7 (15)	10 (8)	127 (5)
Student	36 (2)	7 (1)	7 (3)	4 (9)	3 (2)	57 (2)
Other§	13 (1)	2 (<1)	1 (<1)	1 (2)	0	16 (1)
Region of exposure, no. (%)¶						
Southeast Asia	570 (36)	414 (66)	99 (37)	10 (22)	37 (30)	1,129 (43)
South-Central Asia	406 (26)	146 (23)	21 (8)	3 (7)	22 (18)	598 (23)
Northeastern Asia	217 (14)	13 (2)	25 (9)	0	6 (5)	261 (10)
North Africa	76 (5)	6 (1)	45 (17)	1 (2)	9 (7)	137 (5)
Latin America	121 (8)	15 (2)	7 (3)	21 (46)	10 (8)	174 (7)
Sub-Saharan Africa	55 (3)	18 (3)	16 (6)	1 (2)	16 (13)	106 (4)
Middle East	47 (3)	3 (<1)	38 (14)	0	2 (2)	90 (3)
Eastern Europe	40 (3)	2 (<1)	4 (2)	1 (2)	4 (3)	51 (2)
Western Europe	28 (2)	3 (<1)	6 (2)	4 (9)	5 (4)	46 (2)
Oceania	14 (1)	0	1 (<1)	2 (4)	1 (1)	18 (1)
North America	3 (<1)	1 (<1)	2 (1)	3 (7)	8 (6)	17 (1)
Caribbean	8 (1)	2 (<1)	2 (1)	0	3 (2)	15 (1)
Australia	1 (<1)	0	0	0	2 (2)	3 (<1)

*NHP, nonhuman primate. Data include 4 patients of unknown sex, 6 patients of unknown age, 4 patients of unknown country of residence, 52 patients whose region of exposure was unknown or unable to be ascertained, and 1 patient whose purpose of travel was unknown. †Bear (n = 1), camel (n = 1), *Nasua* spp. coatis (n = 4), cow (n = 1), donkey (n = 2), fox (n = 1), hamster (n = 2), horse (n = 5), human (n = 1), lion (n = 2), mongoose (n = 1), meercat (n = 2), mouse (n = 5), opossum (n = 1), rabbit (n = 2), raccoon (n = 2), rat (n = 12), rodent (n = 4), squirrel (n = 11), tiger (n = 6), and other, unspecified (n = 60). ‡Two patients were exposed to >1 animal; 1 patient was exposed to cat and dog, and 1 patient was exposed to dog and other (tiger). §This category includes immigration (n = 8), medical tourism (n = 8), and military (n = 1). ¶For explanation of GeoSentinel Surveillance Network regions, see Figure 2 (http://www.cdc.gov/mmwr/preview/mmwrhtml/ss6203a1.htm).

The region in which the animal exposure occurred was recorded for 2,645 (98%) patients; most exposures occurred in Southeast Asia (42%), followed by other regions in Asia (32% for south-central and northeastern combined), Africa (9% for North Africa and Sub-Saharan Africa combined) and Latin America (7% for Central and South America combined, including Mexico) (Table 1). Although 42% of all exposures occurred in Southeast Asia, two thirds of all exposures to NHPs occurred there. A very small proportion of patients were exposed to animals in North Africa (5%) and the Middle East, (3%), but 17% and 14% of patients in those regions, respectively, were exposed to cats. Almost half of all bat exposures occurred in Latin America, whereas only 6% of patients overall were exposed there.

The country with the highest proportion of animal exposures was Thailand, followed by Indonesia, Nepal, China, and India (Table 2). Indonesia ranked first for NHP- and bat-related exposures. Among the top 5 countries for cat-related exposures were Turkey and Algeria, and among the top 5 countries for bat-related exposure, 4 were in Latin America (French Guiana, Peru, Mexico, and Suriname).

**Table 2:** Countries with 5 highest levels of exposure among 2,697 patients who sought care for animal exposure and received rabies postexposure prophylaxis at GeoSentinel Surveillance Network sites, January 1997–December 2012, by animal species

No.	Animal, country of exposure, no. (%) exposures					
	Dog, n = 1,618	NHP, n = 638	Cat, n = 271	Bat, n = 46	Other, n = 126	Total, n = 2,697
1	Thailand, 294 (18)	Indonesia, 200 (31)	Thailand, 59 (22)	Indonesia, 7 (15)	Thailand, 16 (13)	Thailand, 534 (20)
2	Nepal, 198 (12)	Thailand, 166 (26)	Turkey, 31 (11)	French Guyana, 5 (11)	India,10 (8)	Indonesia, 314 (12)
3	China, 197 (12)	Nepal, 82 (13)	China, 25 (9)	Peru, 4 (9)	Indonesia, 10 (8)	Nepal, 295 (10)
4	India, 124 (8)	India, 43 (7)	Indonesia, 17 (6)	Mexico, 3 (7)	China, 6 (5)	China, 241 (9)
5	Indonesia, 80 (5)	Vietnam, 21 (3)	Algeria, 15 (6)	Surinam, 3 (7)	Nepal, 6 (5)	India, 185 (7)

### Seasonality

**Figure 1:**
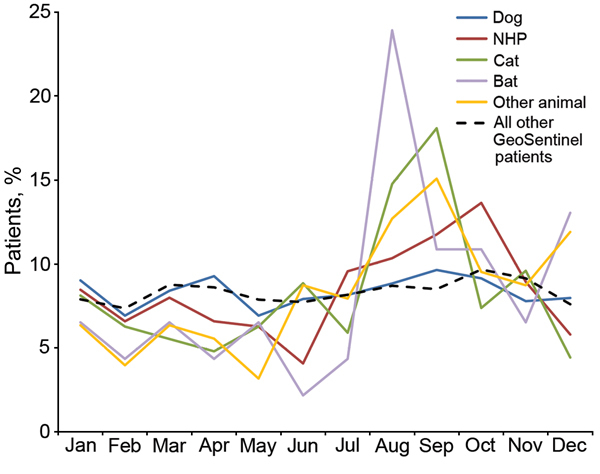
Monthly distribution of animal-related exposure cases requiring rabies postexposure prophylaxis, by exposure species, according to date of initial visit to GeoSentinel clinics, 1997–2012.

Overall, 801 (30%) patients receiving rabies PEP after an animal-related exposure received care at a GeoSentinel site during July–September (Figure 1). This seasonal pattern was most pronounced for those exposed to cats or bats. This finding is in contrast to all patients entered into the GeoSentinel database during the period of study with any diagnosis, 25% of whom received care during July–September.

### Duration of Travel

Travel duration could be determined for 2,452 patients. Among these, median duration was 15 days (range 1–6,205 days) among 1,961 patients who sought care for an animal-related exposure after travel and 20 days (range 1–794 days) among 491 who sought care during travel.

### Trends among Patients Receiving PEP 

**Figure 2:**
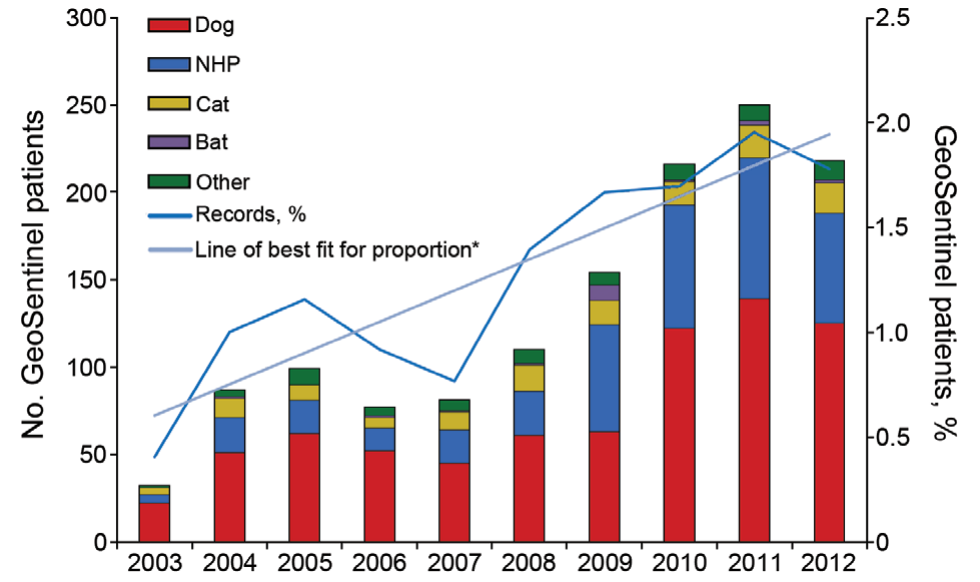
Number of patients requiring rabies postexposure prophylaxis for animal-related exposure, by exposure species and by year and line of best fit for proportion of all GeoSentinel records accounted for by animal-related exposure requiring postexposure prophylaxis, 2003–2012. Limited to patients treated at GeoSentinel sites that were active contributors for the entire listed period. NHP, nonhuman primate. *Linear regression was used to calculate a line of best fit of y = 0.0015x + 0.006.

Of the 2,697 reported animal exposures, 83% occurred during 2007–2012. Among the 138,433 patients who sought care during 2003–2012 at sites that were active GeoSentinel members for that entire period, 1,490 (1.1%) received rabies PEP after an animal exposure at 23 continuously reporting sites. In this group, the proportion of animal-associated exposures relative to total reports to GeoSentinel increased ≈0.1% per year over the 10-year period (β = 0.00149, 95% CI 0.00088–0.00210, p<0.001); the number of animal-associated exposures reported to GeoSentinel in 2012 was 4-fold greater than the number reported in 2003 (Figure 2).

### Rabies Diagnoses

During the study period, 3 patients included in the GeoSentinel database received a diagnosis of rabies (Table 3). All 3 patients died.

**Table 3:** Characteristics of 3 patients with travel-associated rabies, GeoSentinel Surveillance Network, January 1997–December 2012

Year (reference)	Age, y/sex	Citizenship	Reason for travel	Country (source) of exposure
2006 (*2,7,8*)	65/M	Japan	Business (expatriate)	Philippines (dog bite)
2012 (*2,9,10*)	41/M	Canada	Unknown	Island of Hispaniola (unknown)*
2012 (*2,11*)	34/M	Israel	Tourism	India (unknown)

*Although this patient’s place of exposure was initially described as being the Dominican Republic, the exact location or source of exposure could not be definitively determined; Hispaniola comprises Haiti and the Dominican Republic.

## Discussion

Our analysis is a comprehensive survey addressing the epidemiology of animal-related exposures leading to rabies PEP among international travelers. The number of patients (2,697), duration of the study (16 years), and multicenter design (45 sites) provided robust data for this analysis. We found a small but significant rise in the proportion of travelers who sought care at GeoSentinel sites during 2003–2012 and who required rabies PEP, even after we eliminated the bias of increased number of sites by including only continuously reporting sites. It is known from World Tourism Organization data that tourist destinations are becoming more diverse (*12*); increased proportions of international tourists are traveling to countries that have emerging and developing economies and where rabies is endemic. Because the trend toward more exotic travel destinations is predicted to continue well into the future (*12*), demand for rabies pharmaceuticals and postexposure wound care among international travelers will probably continue to grow.

During the study period, 2,697 travelers sought care for animal exposure at GeoSentinel surveillance clinical sites and received rabies PEP. The number of travelers who seek rabies PEP is known to be an underestimate of the actual number of travelers exposed. In a recent survey conducted among 7,681 international travelers leaving the Bangkok airport, two thirds of travelers who reported having been bitten by a potentially rabid animal during their trip sought no medical care (*13*). Although exact figures do not exist, it can be supposed that the prevalence of exposure to potentially rabid animals among international tourists is substantial. The fact that 3 rabies diagnoses were entered into the GeoSentinel database during the study period confirms this supposition. More data defining the epidemiology of rabies exposure and disease among travelers are needed.

Our survey findings confirm those from an earlier GeoSentinel survey conducted among only 320 returned travelers (*14*). However, the inclusion criteria for the 2 analyses differed; the earlier analysis was conducted only among patients who sought care after travel, and 34% were patients who reported an animal exposure but did not receive rabies PEP. Additionally, the structure of the GeoSentinel network has evolved over time (*6*), so cautious interpretation of the comparison is warranted.

Most persons who report to GeoSentinel sites and require rabies PEP are young adult (15–44 years of age) tourists traveling from high-income regions to visit low- and low-middle–income regions. This profile corresponds to the overall traveler population seen at GeoSentinel sites (*15*). This apparent lack of distinction is important because the demographic characteristics of travelers exposed to potentially rabid animals did not differ from those of other ill travelers who seek medical care, which makes it challenging to identify specific travelers who might benefit from reinforced rabies pretravel preventive counseling. Although previous studies have found children to be at highest risk for animal bites requiring PEP (*1*), our results suggest that young adults may also be vulnerable and may also benefit from preventive counseling. We observed a small but significant seasonal pattern (especially for bat and cat exposures), which might be used to guide the pretravel advice given to summer tourist travelers.

The short median duration of travel (2 weeks) among returned travelers consulting for rabies PEP corroborates the World Health Organization recommendation that a travelers’ assessment for risk of an animal bite should not be influenced by the duration of travel (*1*). Our results, however, are not consistent with the current CDC recommendations that preexposure rabies vaccine recommendations should be based, at least in part, on longer durations of stay (*16*), a position that is shared by many countries (*3*). Additionally, among those seeking care at GeoSentinel sites during travel, exposure occurred within a median of 3 weeks of arriving in the country of exposure, which suggests that rabies vaccination may also be indicated for patients embarking on shorter trips.

Of travelers consulting for rabies PEP at a GeoSentinel site, 70% had been exposed while in Asia, most in Southeast Asia. Rabies is endemic to most countries in Asia (*17*). Of 10 patients, 6 were exposed in 5 countries (Thailand, Indonesia, Nepal, China, and India). Large numbers of human rabies cases among the local population are reported from these 5 countries, with the exception of Thailand (Technical Appendix), where only sporadic cases of rabies in humans are now reported (*1*,*17*–*25*; J.M. Shresta, 2012, pers. comm.). Rabies cases in humans are reported from almost all regions in India (rates are highest rates in Chhattisgarh, Uttar Pradesh, and Odisha states) and from almost all regions in China (rates are highest in Guizhou, Guangdong, Hunan, Guangxi, and Guangdong Provinces). In Thailand, rabies cases in humans show no specific geographic distribution. In Indonesia and Nepal, cases are concentrated in specific areas (*1*,*17*–*25*; P. Rupali, 2012, pers. comm.). Travel-associated rabies cases have been reported from all these countries except Indonesia; most such cases were acquired in India and China (*2*). Updated data about the incidence of rabies in many countries is difficult to find, which indicates a need for improved human rabies surveillance (*1*).

For travelers to these 5 countries, rabies vaccine is more accessible than rabies immunoglobulin. Tissue-cultured vaccine is locally produced (China and India) or imported (all 5 countries) and may be available in most cities. Equine rabies immunoglobulin is available from most public hospitals in China and Thailand but may be difficult to find in smaller hospitals, notably in remote rural areas. In India, Indonesia, and Nepal, equine rabies immunoglobulin may be available from large cities only. Human rabies immunoglobulin is generally unavailable except in limited circumstances and at specialized centers (*26*–*32*; P. Rupali, 2012, pers. comm.). All recent studies addressing rabies PEP management in exposed travelers indicate that <1 in 10 travelers received rabies immunoglobulin in the country of exposure (*33*–*36*). In this study, among those who received rabies immunoglobulin after returning to their home country, there was a substantial delay between exposure and administration of rabies immunoglobulin. Some exposed travelers returned home to clinics in their own countries, having received the first dose of vaccine—without rabies immunoglobulin—in the country of exposure >7 days earlier; at this time, administration of rabies immunoglobulin may have reduced benefits. Equine rabies immunoglobulin carries a very low risk for anaphylaxis and is safe and effective (*37*); travelers should be encouraged to accept it when available and prescribed.

Although few patients in our analysis were exposed while in Vietnam or Philippines, these rabies-endemic countries are among the top 10 tourist destinations in Asia (*17*), so travelers to these countries should also be informed about potential rabies exposure and benefits of pretravel vaccination. Given the complex mix of high travel volumes, rabies endemicity, and inconsistent availability of rabies pharmaceuticals, Asia may be a region of considerable rabies risk for travelers.

Although dogs remain the leading animal responsible for exposure among travelers, NHPs account for one quarter of the exposures among patients seen at GeoSentinel sites; this proportion is even higher among tourists, female travelers, and travelers to Southeast Asia. Although rabies cases do occur in NHPs, they are less frequently reported in the literature than are cases in humans. The occurrence of documented transmission of rabies virus from NHPs to humans suggests that rabies PEP is indicated for patients exposed to NHPs in rabies-enzootic countries (*38*).

As found in previous studies (*3*,*4*), we found that a substantial proportion of exposed travelers did not receive pretravel advice. Our data also suggest that only a small proportion had received preexposure rabies vaccination. However, vaccination data were missing for many patients. Public health professionals should work toward increasing the proportion of travelers who receive pretravel medical care, including a selective proportion who receive preexposure rabies vaccination.

This analysis has limitations. The GeoSentinel Surveillance Network captures data only for persons who visit specialized travel or tropical medicine clinics after travel for a travel-related illness or concern; these data do not represent all international travelers. GeoSentinel Surveillance data cannot be used to calculate absolute risk. The composition of travelers included in this analysis probably overrepresents persons traveling to or residing in Asia, as well as those residing in Australia, and underrepresents those residing in North America or traveling to Latin America. Children may also have been underrepresented. In addition, generalizability could be affected by site-specific differences in referral patterns, clinic volumes, patient populations, and travel destinations.

Encouraging travelers to undergo a pretravel risk assessment and prevention counseling may help identify persons who will be at higher risk for a rabies exposure when traveling. The pretravel consultation should educate and warn higher risk travelers to rabies-endemic regions in Asia, Africa, and Latin America about their destination- and itinerary-specific rabies risk profile and the need to avoid contact with animals, notably dogs, NHPs, and cats. Pretravel vaccination against rabies is expensive in many countries (*37*), although the long-lasting resulting immunity may make this investment attractive for some patients in light of cumulative risk from iterative travels (*39*). Schedules of less expensive intradermal preventive vaccination are recommended by the World Health Organization, for travelers as well as others. Several preliminary studies have shown shorter, less expensive preexposure vaccine sched­ules to be effective in several preliminary studies (*37*,*40*). However, they are not yet widely available to travelers, and further large-scale studies are needed before any recommendation can be made. Travel-health specialists should work to identify those for whom pretravel vaccination is most strongly indicated on the basis of risk characteristics. Travelers to rabies-endemic regions, particularly those in Asia, should be well educated about their potential rabies exposure, the importance of avoiding contact with animals, and the potential benefit of pretravel rabies vaccination, regardless of travel duration and traveler demographics.

**Technical Appendix.** Overall rabies epidemiology among humans and availability of rabies biologicals in 5 countries with highest potential for rabid animal–related exposures among travelers.
