# Correction: Vol. 21, No. 4

**DOI:** 10.3201/eid2105.C12105

**Published:** 2015-05

**Keywords:** errata, erratum, correction

The Continuing Medical Education quizzes for 2 articles were inadvertently omitted from the April issue. The quizzes are printed at the back of this issue, and the complete articles and quizzes are available online (http://wwwnc.cdc.gov/eid/article/21/4/14-1479_intro and http://wwwnc.cdc.gov/eid/article/21/4/14-1033_intro).

