# Supplementary material for: Animal-Associated Exposure to Rabies Virus among Travelers, 1997–2012
Source: Emerg Infect Dis. 2015 Apr;21(4):569–77. doi: 10.3201/eid2104.141479 (PMC4378464; doi:10.3201/eid2104.141479)
Supplement: Supplementary file 1 — Technical Appendix. Overall rabies epidemiology among humans and availability of rabies biologicals in 5 countries with highest potential for rabid animal–related exposures among travelers. [file 14-1479-Techapp-s1.pdf]

Article DOI: http://dx.doi.org/10.3201/eid2104.141479

Animal-Associated Exposure to Rabies Virus among Travelers, 1997–2012

Technical Appendix

Technical Appendix Table. Overall rabies epidemiology in humans and availability of rabies biological in top 5 countries for potentially rabid animal–related exposures in travelers

Variable	China	India	Thailand	Indonesia	Nepal
Endemicity	Endemic, reported from almost all regions, with the highest rates in Guizhou, Guangdong, Hunan, Guangxi and Guangdong provinces	Endemic, reported from almost all regions, with the highest rates in Chhattisgarh, Uttar Pradesh and Odisha states	Sporadic, still reported from all regions, including Bangkok. Eradication targeted by 2020	Endemic in Sulawesi, outbreaks in various Islands	Endemic, reported from all regions with high number of cases from the border of India
Human deaths in local population (official reporting)	1524 deaths/year (1996-2008) 1106 deaths (2013)	20,000 deaths (2003)	370 deaths (1980) < 10 deaths/year (2011-2012)	80 deaths/year (1990-1994) 113 deaths (Flores outbreak 1998-2002) ; > 22 deaths (Ambon outbreak, 2003), 216 deaths (Esat Nusa Tenggara outbreak, 1997-2001); >160 deaths (Bali outbreak, 2008-ongoing)	200 deaths/year (2007-2011) Including 32 deaths/year reported from hospitals (2004-2010)
Human deaths in local population (WHO estimation)	7,450 deaths (2010)	16,450 deaths (2010)[1]	-	-	-
Vaccine and current protocol	Tissue-cultured vaccine, locally produced according to WHO standards, may be available in most cities Zagreb intramuscular and Essen intramuscular	Tissue-cultured vaccine (imported and locally produced according to WHO standards), may be available in most cities Essen intramuscular	Tissue-cultured vaccine (imported and locally produced according to WHO standards), may be available in most cities Thai Red Cross intradermal and standard Essen intramuscular regimen	Tissue-cultured vaccine (imported) Zagreb intramuscular, may be available in most cities, but shortage may occur	Tissue-cultured vaccine (imported) may be available in big cities Zagreb intramuscular and Essen Intramuscular
Ig	Locally produced HRIG or ERIG available in most urban areas; imported HRIG generally unavailable except for expatriate medical facilities in Shanghai and Beijing	Available from all major cities	Available in most public hospitals, limited availability in rural area	Limited availability, most exposed travelers did not received RIG	Available from Kathmandu (ERIG) and certain specialized travel clinics (imported HRIG)
Rabies deaths in travelers (1990-2012)	4	10	1	0	2

*WHO, World Health Organization; HRIG, human rabies immunoglobulin; ERIG, equine rabies immunoglobulin; RIG, rabies immunoglobulin.
